# Aquatic moss by-products offer an alternative to algal biomasses for phytostimulant bioproduction

**DOI:** 10.3389/fpls.2026.1988476

**Published:** 2026-10-16

**Authors:** Chiara Anglana, Piergiorgio Capaci, Egidio Stigliano, Lenka Plačková, Karel Doležal, Fabrizio Barozzi, Gian Pietro Di Sansebastiano

**Affiliations:** 1 DiSTeBA (Department of Biological and Environmental Sciences and Technologies), University of Salento, Lecce, Italy; 2 Green Greener srl, Policoro, Italy; 3 Laboratory of Growth Regulators, Faculty of Science, Palacký University and Institute of Experimental Botany Czech Academy of Sciences, Olomouc, Czechia; 4 Department of Chemical Biology, Faculty of Science, Palacký University, Olomouc, Czechia

**Keywords:** abiotic stress, aquatic moss, biostimulants, by-products, *Leptodictyum riparium*, phytohormones, *Taxiphyllum barbieri*, temporary immersion bioreactor (TIB)

## Abstract

**Introduction:**

Aquatic mosses are recently gaining attention for commercial use, stimulating the development of new biomass cultivation approaches. This study explores the potential of by-products from aquatic moss propagation as natural plant biostimulants, addressing the need for low-waste production and sustainable agricultural strategies.

**Methods:**

Using Temporary Immersion Bioreactors, *Leptodictyum riparium* and *Taxiphyllum barbieri* were cultivated, yielding solid non-viable biomass and liquid spent culture medium capable of offering a convenient and eco-sustainable alternative to similar products from algal and plant biomasses. Chemical analyses were performed to identify key plant hormones, including cytokinins (e.g., cis-zeatin, N^6^-isopentenyladenosine), auxins (indole-3-acetic acid, 2-oxindole-3-acetic acid), and abscisic acid (ABA). To evaluate efficacy, *in vitro Nicotiana tabacum* plants were grown under optimal, salt, and osmotic stress conditions and treated with various moss-derived preparations.

**Results:**

Cytokinins dominated the extract fraction with higher concentrations than auxins in both moss species. Cytokinin totals in the extracts were up to three orders of magnitude above the minimum threshold typically required for cytokinin-responsive phenotypic changes. In some cases, auxin concentrations approached, though remained below, the ~100 nM threshold commonly cited for bioactivity. Under optimal conditions, *T. barbieri* extract at 0.1% was the most effective treatment, increasing root length by up to 49% and shoot fresh weight by 93%. Under salt stress, *L. riparium* culture water applied at 3% of medium induced the strongest response, enhancing root length and biomass by up to 260% and 301%, respectively. Under osmotic stress, moderate biomass increases were observed, reaching 94% with *L. riparium* by-product and 48% with *T. barbieri* extract.

**Discussion:**

Overall, biostimulant efficacy was species- and matrix-dependent, with extracts mainly promoting growth under optimal conditions and culture water mitigating salinity stress. These findings indicate that moss-derived by-products are promising candidates for the development of innovative plant biostimulant formulations within a circular and low-input production framework, offering an alternative to other phytohormone-rich biomasses.

## 1 Introduction

Current strategies to improve agricultural productivity emphasize sustainability and nutritional quality (Hebinck et al., 2021). Among these, plant biostimulants, particularly those of natural origin, have gained prominence for enhancing crop performance and climate resilience. They are increasingly employed as growth promoters and pre-stress conditioners in agricultural systems (Yakhin et al., 2017). Both the EU Regulation 2019/1009 and the European Biostimulants Industry Council (EBIC) define plant biostimulants as substances and/or microorganisms that stimulate plant nutritional processes independently of nutrient content, enhancing nutrient use efficiency, abiotic stress tolerance, crop quality, or nutrient availability in the rhizosphere. This functional definition focuses on product performance rather than composition or mode of action, acknowledging that a single ingredient may exert multiple effects.

Biostimulants encompass various categories, including humic and fulvic acids, protein hydrolysates, amino acids and nitrogen-containing compounds, seaweed extracts, plant extracts, inorganic compounds, chitosan and other biopolymers, microorganisms, and industrial by-products (Calvo et al., 2014; du Jardin, 2015; Xu and Geelen, 2018; Bisht and Chhabra, 2024). Within a circular economy framework, industrial by-products have gained increasing attention for their potential to be repurposed as cost-effective and sustainable sources of biostimulant compounds. These include organic residues from agriculture, food processing, aquaculture, and urban waste, rich in bioactive molecules such as amino acids, phenolics, and phytohormones (Xu and Geelen, 2018; Zhang et al., 2024). Examples range from hydrolysates derived from fish waste and poultry feathers to extracts from vegetable peels, compost, and vermicompost, many of which enhance plant growth and stress resilience (Hossain et al., 2017; Etemadian et al., 2021). Their diverse composition and complex mechanisms of action necessitate a multidisciplinary approach to fully elucidate their biostimulant potential (Zhang et al., 2024).

Plant growth regulators (PGRs), including natural phytohormones, are key components in many biostimulant formulations due to their ability to modulate plant growth and stress responses. Phytohormones such as auxins, gibberellins, cytokinins, abscisic acid, jasmonates, and salicylic acid play crucial roles in cell division, elongation, seed development, and defense regulation (Jiang and Asami, 2018; Müller and Munné-Bosch, 2021; Asif et al., 2022). Their presence in natural ingredients, such as seaweed extracts, has been widely documented and contributes significantly to the bioactivity of these materials (Prasad et al., 2010; Ghaderiardakani et al., 2019). Although they have no nutritional value, these compounds effectively regulate growth at low doses and are increasingly being integrated into biostimulant formulations due to their efficacy (Scacchi et al., 2009; Yalçın et al., 2019).

Seaweed extracts are widely used as biostimulants because they have high phytohormone content and, because of that, good efficacy (Chiaiese et al., 2018). Asia dominates global farmed seaweed volume (97%) and Europe has marginal role on the Atlantic coasts, mainly concentrating on Ascophyllum nodosum-based extracts (e.g., the widely commercialized AMPEP biostimulant) (Gupta and Msuya, 2025). The risk of algal natural beds overexploitation raises sustainability concerns (Mac Monagail et al., 2017), on the other hand, cultivating macroalgae outside of their natural environment showed to be complex issue (Campbell et al., 2019).

Surprisingly, relatively few studies have focused on using other plant biomass sources for phytohormone extraction or biostimulant production (Tudu et al., 2022; Espinosa-Antón et al., 2025). Wally et al. (2013) used highly sensitive UPLC-ESI-MS/MS to quantify plant hormones across twelve commercial seaweed extracts (from *Ascophyllum* sp., *Ecklonia* sp., *Macrocystis* sp., *Durvillaea* sp., and *Sargassum* sp.), finding that hormone levels were consistently very low (picomole per gram dry weight) and highly variable between products.

Higher plant-derived biomasses (for example garlic, moringa, alfalfa, etc.) contain auxins (IAA, indole-3-pyruvate), gibberellins, and cytokinins (trans-zeatin and its riboside) at detectable but variable and generally low concentrations that shift depending on plant species, extraction method (Martínez-Lorente et al., 2024). The minimum thresholds known to trigger phenotypic responses in plants are close to 100 nM for auxin, 10 nM for cytokinin, and 100 nM for brassinosteroids but extract content fell far below these physiologically active levels, discouraging exploitation.

More than 50 years of research have demonstrated that the model moss *Physcomitrium patens* produces most known phytohormones, regulating its development in response to both biotic and abiotic stresses. The biosynthesis and signaling pathways of the three classical hormones (auxins, cytokinins, and abscisic acid) are best characterized in this moss model, whereas little is known about other moss species, particularly aquatic ones (Guillory and Bonhomme, 2021). Aquatic mosses represent an interesting case, as these organisms may be more prone to secreting biologically active compounds for agronomic purposes. The interest in investigating the potential of aquatic mosses extracts and by-products arose from the growing interest in aquatic mosses cultivation in controlled conditions for bioremediation and other applications (Papadia et al., 2020; De Matteis et al., 2021; Amitrano et al., 2025; Del Piano et al., 2026).

To produce mosses biomass, Temporary Immersion Bioreactors (TIBs) proved to represent a valid technology. TIBs are advanced plant culture systems widely used in modern biotechnology for micropropagation and beyond; their simple design and operational flexibility have led to the development of several variants adapted for secondary metabolite research, molecular farming, and phytoremediation of toxic compounds (Steingroewer et al., 2013).

Compared to conventional solid medium systems (SCM) and continuous liquid-phase bioreactors, TIB offer a dynamic environment that enhances gas exchange, optimizes nutrient uptake, and reduces physiological disorders such as hyperhydricity or tissue asphyxiation (Steingroewer et al., 2013; Yaseen et al., 2013; Anglana et al., 2023). Several studies have shown that TIBs promote higher proliferation rates and result in superior plantlet quality (Capaci et al., 2024, 2026). Moreover, TIB offer a high degree of compatibility with automation, making them especially suitable for large-scale production and significantly lowering labor costs (Georgiev et al., 2014; De Carlo et al., 2021).

Finally, TIBs have emerged as highly effective platforms for producing valuable plant-derived secondary metabolites, including alkaloids, flavonoids, and phenolic compounds (Quiala et al., 2006). Their efficiency in this context is largely attributed to their ability to maintain sterile (axenic) culture conditions while simultaneously promoting the targeted growth of plant organs rich in these bioactive compounds. The system’s design eliminates the need for pesticides and herbicides, thereby ensuring the production of uncontaminated metabolites.

The propagation of aquatic mosses such as *Leptodictyum riparium* and *Taxiphyllum barbieri* in TIB has recently been shown to significantly enhance biomass production compared to traditional liquid culture in flasks (Anglana et al., 2024). This cultivation method produces two types of by-products: a solid fraction, consisting of fragmented or nonviable moss biomass, and a liquid fraction, the spent culture medium. The solid biomass, obtained through qualitative selection and separation from viable gametophytes, was subjected to a simple aqueous extraction procedure. The liquid medium, having interacted with the mosses over time, contained secondary metabolites, including phytohormones and signaling molecules. Liquid medium and biomasses aqueous extracts were tested for their biostimulant effect and phytohormones were quantified. The aim of this work is to show that the by-products produced during propagation of aquatic mosses are valuable resources for phytohormone bioproduction and their direct biostimulant activity for agricultural use.

## 2 Materials and methods

### 2.1 Axenic *in vitro* propagation of aquatic mosses

Axenic cultures of *Leptodictyum riparium* and *Taxiphyllum barbieri* (provided by Green Greener srl, Policoro, MT, Italy; https://www.greengreener.net/) were established in liquid cultures in shaken (130 rpm) Erlenmeyer flasks containing sterilized deionized water (60 µS/cm). Temporary immersion bioreactor (TIB) technology was also used, with Setis™ bioreactors filled with either deionized water, deionized water supplemented with 5% PpNO_3_ or PpNH_4_ medium, or full-strength PpNO_3_ or PpNH_4_ medium (Ashton et al., 1979). Immersion time was 2 min every 6 h. Cultures were maintained at 22 ± 2 °C, under a 16/8 h light/dark photoperiod and a light intensity of 150 µmol m^-2^ s^-1^. Growth rate was determined as the increase in fresh weight (FW) and expressed as relative growth rate (RGR):


$$\text{RGR}=\frac{{\text{lnFW}}_{2}-{\text{lnFW}}_{1}}{t_{2}-t_{1}}$$

where FW_1_ and FW_2_ are moss fresh weights at times t_1_ and t_2_, respectively. For all culture types, biological independent replicates were 6.

### 2.2 Extraction and preparation of moss biomass and by-product

Samples were processed in three different forms. First the fresh biomass discarded from selection at the end of the growth cycle was freeze-dried to perform accurate extraction and analyze their plant hormone content. Second, the discarded biomass (10 g) was extracted in 500 mL of RPE water using an autoclave process with a pressure of 1 atm applied at 120 °C for 16 minutes. The extract was filtered through a 0.2 μm filter. Third, the spent culture medium (1 L of water) in which 30 g of moss had been grown for 1 month in TIB systems to be considered a waste by-product cyclically generated after the culture cycle. It was briefly centrifuged to eliminate solid particles and filtered directly through a 0.2 μm filter. The extracts and spent medium by-product were analyzed for plant hormone content and tested for biostimulant properties.

### 2.3 Qualitative and quantitative analysis of growth regulators

Freeze-dried samples were processed in three technical replicates, each weighing approximately 0.5 mg dry weight (DW) for cytokinins (CK) analysis, and liquid samples of 0.5 mL for CK and 0.3 mL for auxins. In-tip μSPE was used as a miniaturized method for the purification of CK metabolites and for auxins plus ABA. CK samples were extracted in 0.5 mL modified Bieleski buffer (60% MeOH, 10% HCOOH, 30% H_2_O) together with a mixture of isotope-labeled CK internal standards (0.2 pmol per sample for B, R, 7G, 9G and 0.5 pmol per sample for OG, NT) to verify purification recovery and validate quantification (Hauserová et al., 2005). Samples were purified using StageTips containing three layers of different stationary phases (C18, SDB-RPS, Cation Attract SPE) following the protocol described by Svačinová (Svačinová et al., 2012). The eluates were dried and dissolved in 30 μL of 10% MeOH. Samples were separated on a reversed-phase column (Acquity UPLC^®^ BEH Shield RP18, 1.7 μm; 2.1 × 150 mm, Waters) and analyzed by UHPLC–MS/MS (Acquity UPLC^®^ I-class system; Waters, Milford, MA, USA) coupled to a triple quadrupole mass spectrometer with an electrospray interface (Xevo TQ-S, Waters, Manchester, UK) using the method described in Svačinová et al. (2012). Quantification was obtained by multiple reaction monitoring (MRM) of [M+H]^+^ ions and the appropriate product ion. For each CK metabolite, optimal conditions, dwell time, cone voltage, and collision energy were optimized in the collision cell (Novák et al., 2008; Svačinová et al., 2012). The ratio between endogenous CK and the known concentration of added isotope-labeled standard was determined and subsequently used to quantify endogenous CK levels (Novák et al., 2008).

For auxins and ABA determination, samples were extracted in 0.3 mL 50 mM Na-phosphate buffer (pH 7.0; 4 °C) containing 0.1% diethyldithiocarbamic acid, and a mixture of isotope-labeled ABA and auxin internal standards (5 pmol per sample) was added (Pěnčík et al., 2009). Extracted samples were acidified with 0.1 M HCl to pH 2.7 before loading onto in-tip μSPE containing three layers of C18 and SDB-XC stationary phases, and the purification protocol was carried out according to Pěnčík et al. (2018). Eluates were dried, dissolved in 30 μL of 10% MeOH, and injected onto a reversed-phase column (Kinetex 1.7 μm C18 100A, 50 × 2.1 mm; Phenomenex) and analyzed using an Acquity UPLC^®^ I-class system (Waters, Milford, MA, USA) coupled to a Xevo™ TQ-S (Waters, Manchester, UK) equipped with an electrospray ionization (ESI) interface following the method described by Pěnčík (Pěnčík et al., 2009). Quantification was performed using MassLynx software with an isotopic dilution standard method.

Concentration data from liquid matrices (by-product and extract) are expressed as pmol mL^-1^, to compare these with data in literature, these were converted directly and exactly to nM:


$$1\text{pmol/mL}=\frac{10^{-12}\text{mol}}{10^{-3}\text{L}}=10^{-9}\text{mol/L}=1\text{nM}$$

In the case of solid biomass matrix, values are expressed as pmol g^-1^ DW considering a normalized quantity, not a solution concentration. This cannot be converted to nM without an assumption about tissue water content (fresh-to-dry weight ratio), which isn’t reported in the manuscript, so native units were maintained rather than fabricate a conversion factor.

### 2.4 *In vitro* experiments to evaluate biostimulant effect of moss propagation by-products

*Nicotiana tabacum* plants were used for the *in vitro* experiments with a number of 40 plants per replica and 3 replicas for experiment. Seeds were surface-sterilized for 5 min in a sodium hypochlorite solution diluted in sterile bi-distilled water (1:5, v/v), rinsed with 250 mL sterile water, and sown on solid ½ Murashige and Skoog (MS) medium containing 0.5% sucrose. Seeds underwent vernalization at 8 °C for 48 h and germinated within 5 days in a growth chamber under continuous light at 24 °C. Seedlings were then transferred to plates with ½ MS agarized medium containing 0.5% sucrose, then placed vertically in a growth chamber (16/8 h light/dark, 21 °C) for 15 days. Treatments were incorporated into the culture medium during plate preparation by mixing the treatment or control solution, sterilized by 0.20 μm filtration, at final concentrations of 0.1%, 0.5%, 1%, 3%, or 5%. The control consisted of sterile water with conductivity of 60 µS/cm. Since no previous information was available for these moss-derived products, the concentrations were selected as an exploratory dose–response range to evaluate potential concentration-dependent effects, following the general approach used for plant biostimulants (Ugena et al., 2018; Ogunsanya et al., 2022).

Treatments included by-product made up of water from *L. riparium* and *T. barbieri* cultured in temporary immersion bioreactors (SETIS™) and extract of both moss (120 °C, 16 min, 1 atm), obtained using waste from biomass propagation. Measured parameters were primary root length, assessed using ImageJ image analysis software, and shoot fresh weight, determined using an analytical balance.

For *in vitro* stress experiments, the same protocol was followed, with additional treatments to simulate abiotic stress. Salt stress was induced by adding 150 mM NaCl to the culture medium, while osmotic stress was simulated with 300 mM mannitol, applied to both treatment and corresponding control plates. The control consisted of sterile water at 60 µS/cm. Treatments under stress conditions included *L. riparium* by-product and extract at 0.1%, 1%, and 3% and *T. barbieri* by-product and extract at 0.1%, 1%, and 3%.

### 2.5 Statistical analysis and figure manipulation

Statistical analyses were performed in R. Prior to hypothesis testing, the core statistical assumptions were formally evaluated for each dataset. Normality of residuals was assessed using the Shapiro–Wilk test, while homogeneity of variances was verified using Levene’s test. Independence of residuals was evaluated via the Durbin–Watson test. To account for variations in variance homogeneity across treatments, a uniform parametric pathway utilizing a one-way Welch’s ANOVA was performed for all datasets, followed by Games–Howell *post hoc* pairwise comparisons. The statistical unit was defined as the individual moss clump or individual *N. tabacum* plant. Multiple comparisons were conducted independently within each specific experiment, and statistical significance was graphically denoted via asterisks exclusively relative to the control condition (water media in case of the mosses), while the complete matrix of all pairwise comparisons is provided in the **Supplementary Data Sheet 1**. Significance levels were indicated as follows: p < 0.05 (*), p < 0.01 (**), p < 0.001 (***), p < 0.0001 (****). Data in text and figures are expressed as mean ± standard deviation (SD). Images were assembled using Adobe Photoshop CS6.

## 3 Results

### 3.1 Propagation of aquatic mosses in temporary immersion bioreactors

Growth of the two moss species in traditional flask cultures and in TIB was compared under nutrient-free conditions and expressed as relative growth rate (RGR). For *L. riparium*, the slower growth in flask culture and the clear advantage of TIB were previously reported (Anglana et al., 2024) and are shown again in **Figure 1A** to allow comparison between the two species.

**Figure 1:**
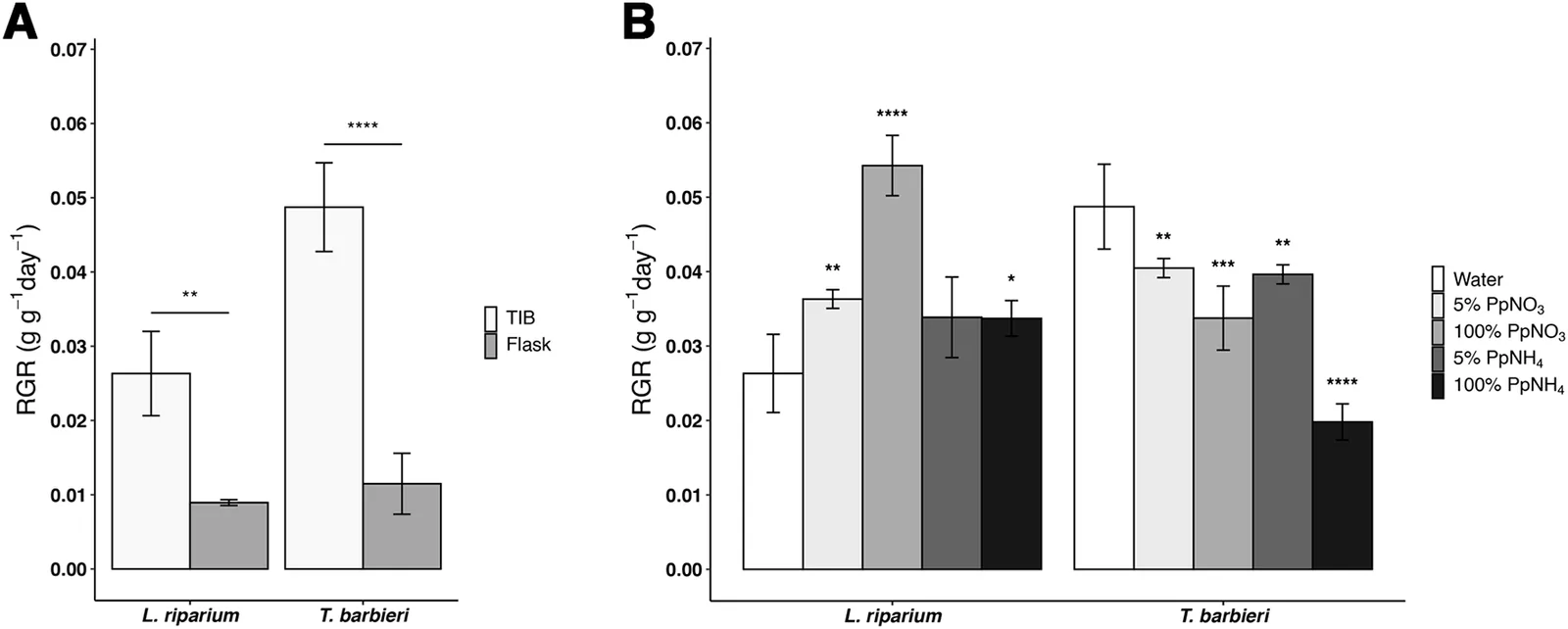
**(A)** Difference, expressed as relative growth rate RGR (g g^-1^day^-1^) of L. riparium and T. barbieri between traditional flask method and TIB (temporary immersion bioreactor) in deionized water (60 µS/cm); **(B)** Growth in TIB of the two mosses, expressed as RGR, using deionized water and different concentrations of growth-medium supplements (5% and 100% PpNO_3_ and PpNH_4_). Statistical analysis was performed using Welch’s ANOVA, followed by Games–Howell *post hoc* tests to adjust p-values for multiple comparisons. Significance levels were indicated as follows: p < 0.05 (*), p < 0.01 (**), p < 0.001 (***), p < 0.0001 (****). In **(B)**, only significant differences relative to the control group (Water) are displayed; the overall Welch’s ANOVA and the complete matrix of Games-Howell *post-hoc* pairwise comparisons are available in **Supplementary Data Sheet 1** (Sheet: **Figure 1**). Error bars displayed in the figures represent the Standard Deviation (SD). Biological independent replicates were n ≥ 6.

*T. barbieri* exhibited a similar pattern of response to the culture system but with markedly higher RGR values, reaching 0.049 ± 0.008 g g^-1^ day^-1^ in TIB and 0.011 ± 0.004 g g^-1^ day^-1^ in flask culture (**Figure 1A**).

To identify the most suitable growth medium for each species, growth was compared in TIB systems under different culture conditions using various media (**Figure 1B**). When cultured in deionized water, *L. riparium* exhibited the lowest RGR (0.026 ± 0.005 g g^-1^ day^-1^), whereas *T. barbieri* showed a higher RGR under the same condition (0.049 ± 0.008 g g^-1^ day^-1^). For *L. riparium*, the optimal medium was 100% PpNO_3_, yielding the highest RGR (0.054 ± 0.004 g g^-1^ day^-1^). Growth was also improved compared to water when cultured in 5% PpNO_3_, 5% PpNH_4_, and 100% PpNH_4_ (0.036 ± 0.001, 0.034 ± 0.005, and 0.034 ± 0.002 g g^-1^ day^-1^, respectively), although none of these conditions surpassed the performance observed with 100% PpNO_3_. In *T. barbieri*, both diluted PpNO_3_ and PpNH_4_ media resulted in moderately reduced RGR values (0.041 ± 0.001 and 0.040 ± 0.001 g g^-1^ day^-1^, respectively). Growth decreased further in 100% PpNO_3_ (0.034 ± 0.004 g g^-1^ day^-1^), and the lowest RGR was recorded in 100% PpNH_4_ (0.020 ± 0.002 g g^-1^ day^-1^).

### 3.2 Chemical characterization of moss biomass, liquid by-product and biomass extract of mosses

Different preparation derived from the two moss species grown in the TIB systems were analyzed for plant hormones, notably: the dry biomass, the by-product of growth liquid medium after 1 month and the extract from biomass. The extraction was performed on 30 g of biomass in 1 L of liquid culture medium consisting of deionized water (60 µS/cm).The following phytohormones were identified via UHPLC-MS analysis: trans-zeatin (tZ), trans-zeatin O-glucoside (tZOG), trans-zeatin riboside (tZR), trans-zeatin riboside O-glucoside (tZROG), cis-zeatin (cZ), cis-zeatin O-glucoside (cZOG), cis-zeatin riboside (cZR), cis-Zeatin riboside O-glucoside (cZROG), cis-zeatin riboside-5′-monophosphate (cZR5’MP), dihydrozeatin riboside (DHZR), N6-isopentenyladenosine (iP), N6-isopentenyladenosine riboside (iPR), isopentenyladenosine-5′-monophosphate (iPR5’MP), meta-topolin riboside (mTR), ortho-topolin riboside (oTR), para-topolin riboside (pTR), kinetin (K), kinetin riboside (KR), indole-3-acetic acid (IAA), 2-oxindole-3-acetic acid (oxIAA), indole-3-acetic acid aspartate (IAAsp) and abscisic acid (ABA).

Zeatin-type cytokinins were detected in samples as a complex set of interconvertible metabolites, including biologically active free bases, carrier ribosides, biosynthetic riboside-5′-monophosphates, and reversibly inactivated O-glucosylated forms, present in both trans- and cis-isomeric configurations. As reported in the **Tables 1**, **2**, the highest value of tZ was found in the extract of *T. barbieri* (17.060 ± 0.166 pmol mL^-1^). The highest tZOG level was found in the biomass of *L. riparium* (6.397 ± 0.561 pmol g^-1^). The other two forms of the phytohormone in trans configurations were present only in *L. riparium* extract and in biomass and extract of *T. barbieri*, with a higher value of tZROG (80.567 ± 1.416 pmol mL^-1^) in this last one.

**Table 1:** Quantitative UHPLC-MS analysis, expressed in pmol g^-1^ for biomass and pmol mL^-1^ for liquid by-products and extract, of plant hormones present in moss *L. riparium*.

Hormones	*L. riparium* biomass	*L. riparium* liquid by-product	*L. riparium* extract
tZ	0.921 ± 0.081	<LOD	10.690 ± 0.009
tZOG	6.397 ± 0.561	<LOD	<LOD
tZR	<LOD	<LOD	13.345 ± 0.168
tZROG	<LOD	<LOD	2.792 ± 0.272
cZ	159.867 ± 24.569	2.183 ± 0.123	47.236 ± 0.399
cZOG	212.639 ± 10.615	<LOD	167.455 ± 4.060
cZR	2.260 ± 0.278	<LOD	367.413 ± 6.598
cZROG	5.219 ± 0.577	<LOD	1848.087 ± 24.766
cZR5’MP	<LOD	<LOD	1454.621 ± 37.639
DHZR	<LOD	<LOD	1.633 ± 0.084
iP	9.639 ± 1.025	0.114 ± 0.001	74.981 ± 0.705
iPR	0.240 ± 0.001	<LOD	32.326 ± 0.310
iPR5’MP	<LOD	<LOD	123.779 ± 2.547
mTR	<LOD	<LOD	1.256 ± 0.140
oTR	<LOD	<LOD	5.778 ± 0.247
pTR	<LOD	<LOD	4.450 ± 0.101
K	<LOD	<LOD	336.775 ± 2.521
KR	<LOD	<LOD	1.007 ± 0.059
IAA	27.76 ± 5.07	<LOD	26.00 ± 0.47
oxIAA	1210.74 ± 142.48	<LOD	21.22 ± 0.12
IAAsp	<LOD	<LOD	0.87 ± 0.01
ABA	<LOD	2.54 ± 0.06	0.63 ± 0.01

tZ, trans-zeatin; tZOG, trans-zeatin O-glucoside; tZR, trans-zeatin riboside; tZROG, trans-zeatin riboside O-glucoside; cZ, cis-zeatin; cZOG, cis-zeatin O-glucoside; cZR, cis-zeatin riboside; cZROG, cis-zeatin riboside O-glucoside; cZR5’MP, cis-zeatin riboside-5′-monophosphate; DHZR, dihydrozeatin riboside; iP, N^6^-isopentyladenosine; iPR, N^6^-isopentyladenosine riboside; iPR5’MP, isopentenyladenosine-5′-monophosphate; mTR, meta-topolin riboside; oTR, ortho-topolin riboside; pTR, para-topolin riboside; K, kinetin; KR, kinetin riboside; IAA, Indole-3-acetic acid; oxIAA, 2-oxindole-3-acetic acid; IAAsp, indole-3-acetic acid aspartate; ABA, abscisic acid. LOD means limit of detection.

**Table 2:** Quantitative UHPLC-MS analysis, expressed in pmol g^-1^ for biomass and pmol mL^-1^ for liquid by-products and extract, of plant hormones present in moss *T. barbieri*.

Hormones	*T. barbieri* biomass	*T. barbieri* liquid by-product	*T. barbieri* extract
tZ	0.083 ± 0.015	<LOD	17.060 ± 0.166
tZOG	1.254 ± 0.026	<LOD	5.754 ± 0.055
tZR	0.454 ± 0.020	<LOD	29.166 ± 0.729
tZROG	9.838 ± 1.468	<LOD	80.567 ± 1.416
cZ	2.182 ± 0.406	0.226 ± 0.005	15.745 ± 0.010
cZOG	128.424 ± 2.109	<LOD	1309.561 ± 27.842
cZR	22.733 ± 7.294	<LOD	721.330 ± 19.427
cZROG	1027.489 ± 116.400	<LOD	14782.133 ± 291.484
cZR5’MP	<LOD	<LOD	733.796 ± 6.634
DHZR	<LOD	<LOD	3.685 ± 0.126
iP	1.455 ± 0.068	0.386 ± 0.008	22.372 ± 0.245
iPR	11.80 ± 1.703	<LOD	106.555 ± 0.819
iPR5’MP	<LOD	<LOD	123.194 ± 2.892
mTR	<LOD	<LOD	1.453 ± 0.069
oTR	<LOD	<LOD	2.700 ± 0.058
pTR	<LOD	<LOD	<LOD
K	<LOD	<LOD	332.738 ± 0.792
KR	<LOD	<LOD	1.485 ± 0.071
IAA	149.23 ± 18.76	<LOD	44.59 ± 0.27
oxIAA	3168.67 ± 160.01	<LOD	30.93 ± 0.57
IAAsp	<LOD	<LOD	2.15 ± 0.01
ABA	<LOD	0.67 ± 0.00	1.17 ± 0.01

tZ, trans-zeatin; tZOG, trans-zeatin O-glucoside; tZR, trans-zeatin riboside; tZROG, trans-zeatin riboside O-glucoside; cZ, cis-zeatin; cZOG, cis-zeatin O-glucoside; cZR, cis-zeatin riboside; cZROG, cis-zeatin riboside O-glucoside; cZR5’MP, cis-zeatin riboside-5′-monophosphate; DHZR, dihydrozeatin riboside; iP, N^6^-isopentyladenosine; iPR, N^6^-isopentyladenosine riboside; iPR5’MP, isopentenyladenosine-5′-monophosphate; mTR, meta-topolin riboside; oTR, ortho-topolin riboside; pTR, para-topolin riboside; K, kinetin; KR, kinetin riboside; IAA, Indole-3-acetic acid; oxIAA, 2-oxindole-3-acetic acid; IAAsp, indole-3-acetic acid aspartate; ABA, abscisic acid. LOD means limit of detection.

Regarding the cis configuration of zeatin, in its free form (cZ), the highest value was observed in the biomass of *L. riparium*, with 159.867 ± 24.569 pmol g^-1^, and was significantly lower in the other species (15.745 ± 0.010 pmol mL^-1^in the *T. barbieri* extract). The concentration of cZOG was consistently higher in *T. barbieri* extract (1309.561 ± 27.842 pmol mL^-1^), followed by biomass of *L. riparium* (212.639 ± 10.615 pmol g^-1^). The highest value of cZR was observed in *T. barbieri* extract with 721.330 ± 19.427 pmol mL^-1^and the same was for cZROG with a value of 14782.133 ± 291.484 pmol mL^-1^). cZR5’MP was detected only in extracts at a higher concentration in *L. riparium* with 1454.621 ± 37.639 pmol mL^-1^; although at lower concentrations, DHZR was still found only in extracts mainly of *T. barbieri* (3.685 ± 0.126 pmol mL^-1^) (**Tables 1**, **2**).

As for the release of zeatin into the liquid medium, only cZ was found, the values were 2.183 ± 0.123 pmol mL^-1^in *L. riparium* and 0.226 ± 0.005 pmol mL^-1^ in *T. barbieri* (**Tables 1**, **2**).

Another plant hormone detected was N6-isopentyladenosine in iP, iPR and iPR5’MP forms. For iP a higher concentration was found in *L. riparium* with 9.639 ± 1.025 pmol g^-1^ in biomass and 74.981 ± 0.705 pmol mL^-1^ in extract. The concentration of iPR was higher in the moss *T. barbieri* with 11.804 ± 1.703 pmol g^-1^in biomass and 106.555 ± 0.819 pmol mL^-1^ in extract. iPR5’MP was found only in two extracts at similar concentration (**Tables 1**, **2**).

In liquid by-products only iP was detected at very low concentrations: 0.386 ± 0.008 pmol mL^-1^in *T. barbieri* and 0.114 ± 0.001 pmol mL^-1^in *L. riparium* (**Tables 1**, **2**).

Other cytokinin classes were detected only in the extracts, among these the topolin derivatives (mTR, oTR and pTR) with oTR being the most abundant in *L. riparium* (5.778 ± 0.247 pmol mL^-1^L). Kinetin was detected both as the free base (K) and as the ribosidic form (KR) with similar K values for the two mosses: 336.775 ± 2.521 pmol mL^-1^for *L. riparium* and 332.738 ± 0.792 for *T. barbieri* (**Tables 1**, **2**).

Auxins IAA, oxIAA and IAAsp were detected in the biomass and in the extracts of the mosses. In particular, IAA was found in higher concentrations in *T. barbieri* biomass with 149.23 ± 18.76 pmol g^-1^. A higher concentration values of oxIAA was found always in *T. barbieri* biomass with 3168.67 ± 160.01 pmol g^-1^, followed by *L. riparium* biomass with 1210.74 ± 142.48 pmol g^-1^. IAAsp was found only in the extracts at low concentrations (**Tables 1**, **2**).

ABA was detected only in the extract and liquid by-product. Specifically, in extract of *L. riparium* at a concentration of 0.63 ± 0.01 pmol mL^-1^and *T. barbieri* at a concentration of 1.17 ± 0.01 pmol mL^-1^. In liquid by-product was found at a concentration of 2.54 ± 0.06 pmol mL^-1^in *L. riparium* and 0.67 ± 0.00 pmol mL^-1^in *T. barbieri* (**Tables 1**, **2**).

### 3.3 Evaluation of the biostimulant effect of moss biomass and its by-product

Mosses were grown in TIBs using sterile water (60 μS/cm) as a growth medium to analyze metabolites produced by the mosses and to identify methods for valorizing their byproducts, including agricultural applications such as plant biostimulation. The fastest-growing moss is *T. barbieri*, while *L. riparium* requires greater quantities of water in proportion to the biomass produced, and wastewater is the most important by-product. The efficient production process of *T. barbieri*, on the other hand, generates a byproduct biomass as a consequence of qualitative selection of the gametophyte. However, both biomass extracts and liquid byproducts from the two moss species were tested.

#### 3.3.1 Effects of liquid by-product and extract of *L. riparium*

The culture medium (liquid by-product) used for the propagation of *L. riparium*, after a one-month growth cycle, was tested *in vitro* on *Nicotiana tabacum* plants, showing notable stimulating effects. Regarding primary root growth in *N. tabacum*, no significant differences were observed compared to the untreated control, except for a slight increase of 8% at the 1% concentration. However, in terms of fresh shoot biomass, excluding the lowest concentration (0.1%) which was similar to the control, all other treatments showed an increase in fresh biomass ranging from 18% to 26%. Considering both parameters, the 1% concentration treatment yielded the best results (**Figure 2A**).

**Figure 2:**
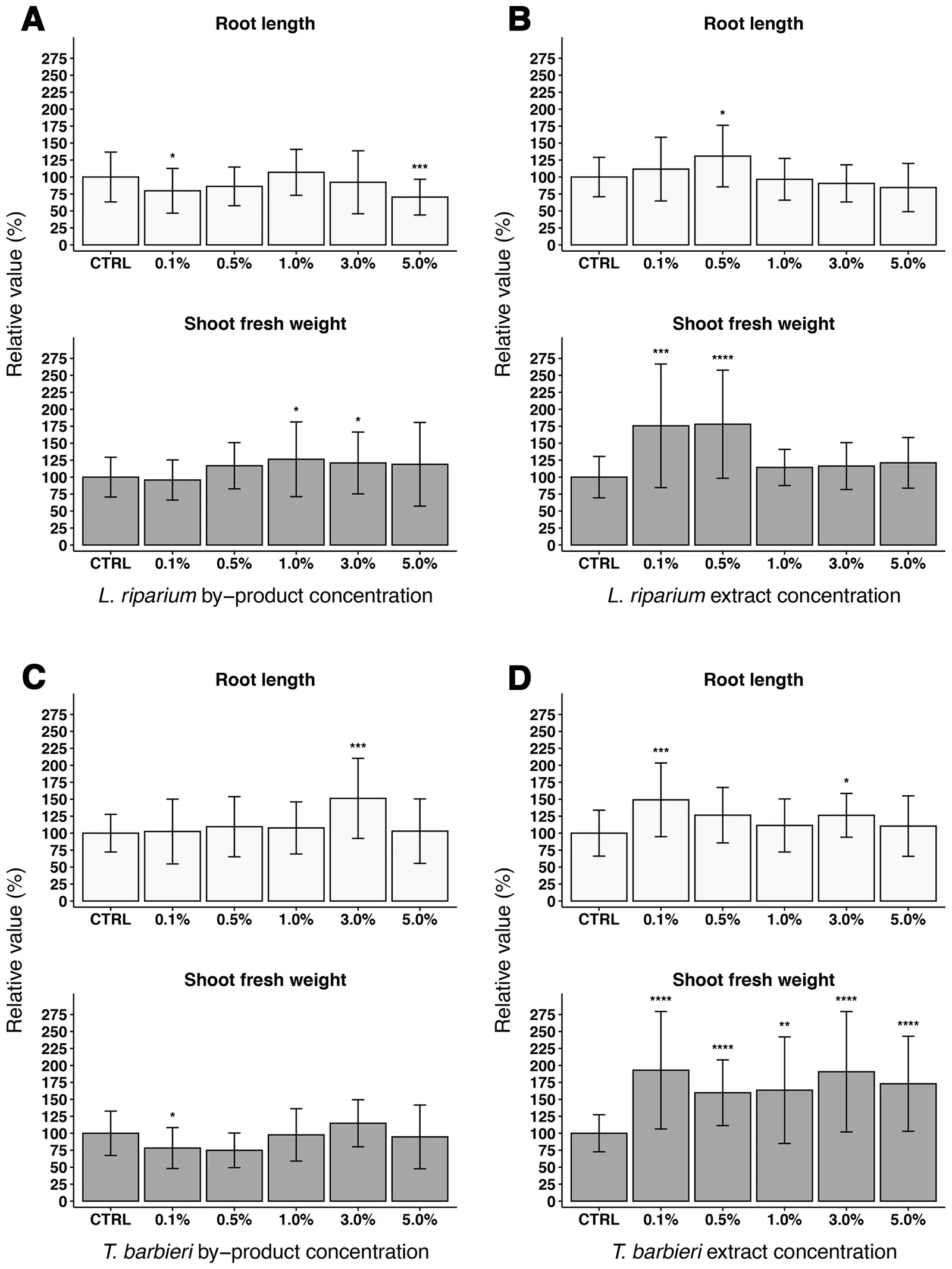
Evaluation of the biostimulant effects of the treatment based on **(A)** by-product of moss *L. riparium*; **(B)** *L. riparium* moss extract; **(C)** by-product of moss *T. barbieri*; **(D)** *T. barbieri* moss extract, added at different concentrations, from 0.1 to 5%, to the culture medium. After 15 days of growth, the length of the primary root and the fresh weight of the aerial part of *N. tabacum* plants were measured. The data are expressed as relative (%) setting the control (CTRL) equal to 100. Statistical analysis was performed using Welch’s ANOVA, followed by Games–Howell *post hoc* tests to adjust p-values for multiple comparisons. Significance levels were indicated as follows: p < 0.05 (*), p < 0.01 (**), p < 0.001 (***), p < 0.0001 (****). In the Figure, only significant differences relative to the control group are displayed; the overall Welch’s ANOVA and the complete matrix of Games-Howell *post-hoc* pairwise comparisons are available in **Supplementary Data Sheet 1** (Sheet: **Figure 2**). Error bars displayed in the figures represent the Standard Deviation (SD). For each analyzed parameter, at least 15 plants per condition were measured within each independent biological replicate, resulting in a total of at least 45 plants analyzed per condition across the three replicates (n = 3).

The waste moss biomass extract in bidistilled water was tested similarly to liquid by-product showing notable stimulating effects. In particular, the extract from *L. riparium* at a concentration of 0.5%, showed an increase in root length of up to 30% and an increase in fresh biomass weight of up to 78% (**Figure 2B**) compared to control.

#### 3.3.2 Effects of by-product and extract of *T. barbieri*

Also, liquid by-product derived from *T. barbieri* was tested *in vitro* on *N. tabacum*. No particular differences were observed in root growth compared to the control, except for the 3% treatment which led to a significant increase of 51%. No significant differences were observed for the fresh weight of the aerial part but an increase of 14% always for the 3% concentration. (**Figure 2C**).

*T. barbieri* biomass by-product extract was tested revealing a positive response across all concentrations compared to the untreated control, with an increase in primary root length up to 49% and fresh biomass up to 93%, being 0.1% the most effective concentration (**Figure 2D**).

#### 3.3.3 Salt stress experiment with by-product and extract of *L. riparium*

Moss liquid by-product and extract were also tested *in vitro* under abiotic stress conditions, simulating salinity stress by adding 150 mM NaCl. Regarding the tests conducted with liquid by-product from *L. riparium*, a particularly marked effect was observed (**Figure 3A**). Except for the lowest concentration, which yielded results similar to the untreated control, the 1% and 3% treatments led to a remarkable recovery of stressed tobacco seedlings compared to the control. Specifically, root length increased by 218% and 260%, while shoot fresh weight increased by 254% and 301%, respectively (**Figure 3A**).

**Figure 3:**
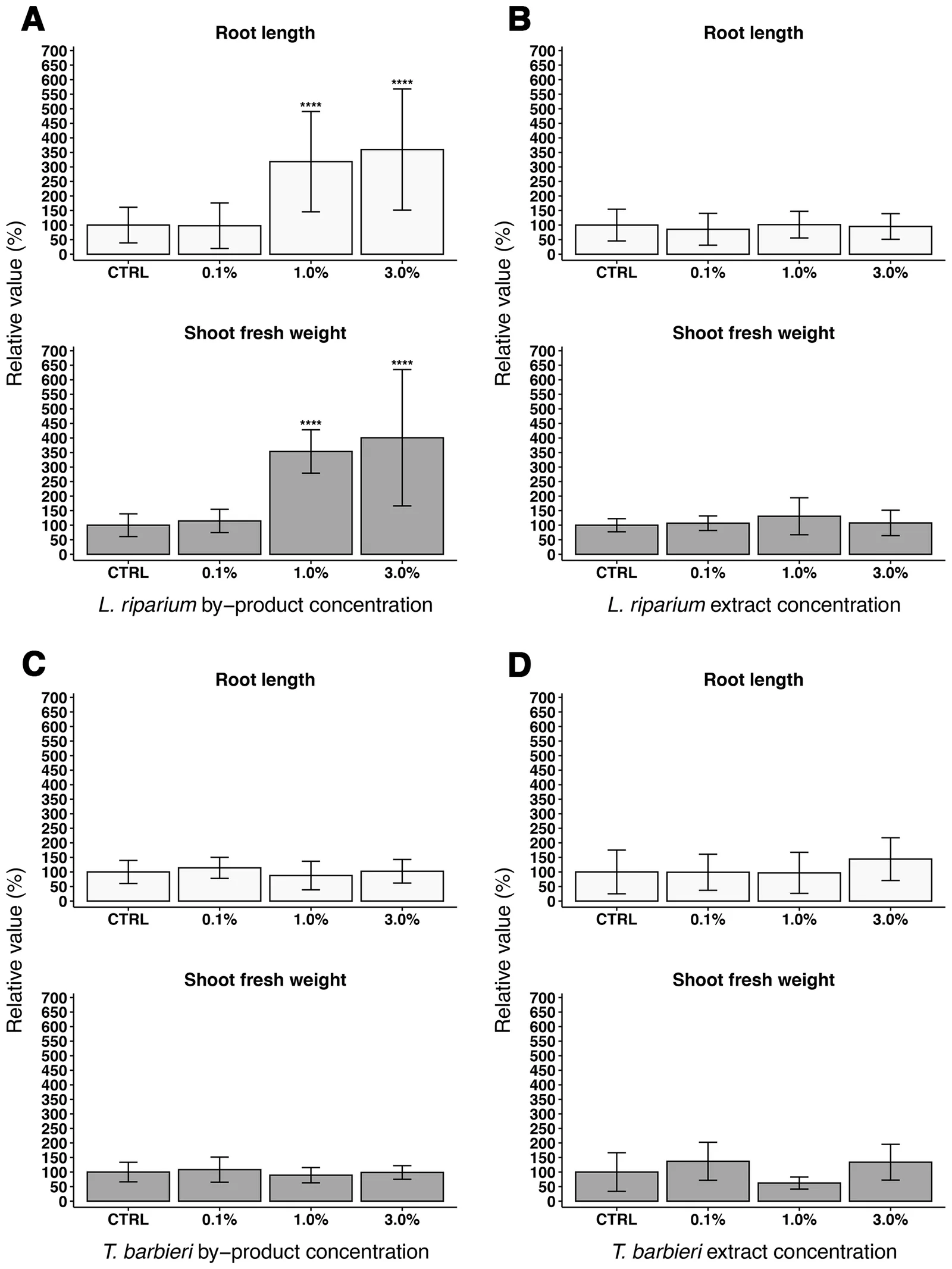
Evaluation of the biostimulant effects under salt stress condition of the treatment based on **(A)** by-product of moss *L. riparium*; **(B)** *L. riparium* moss extract; **(C)** by-product of moss *T. barbieri*; **(D)** *T. barbieri* moss extract, added at different concentrations, from 0.1 to 3%, to the culture medium. After 15 days of growth, the length of the primary root and the fresh weight of the aerial part of *N. tabacum* plants were measured. The data are expressed as relative (%) setting the control (CTRL) equal to 100. Statistical analysis was performed using Welch’s ANOVA, followed by Games–Howell *post hoc* tests to adjust p-values for multiple comparisons. Significance levels were indicated as follows: p < 0.05 (*), p < 0.01 (**), p < 0.001 (***), p < 0.0001 (****). In the Figure, only significant differences relative to the control group are displayed; the overall Welch’s ANOVA and the complete matrix of Games-Howell *post-hoc* pairwise comparisons are available in **Supplementary Data Sheet 1** (Sheet: **Figure 3**). Error bars displayed in the figures represent the Standard Deviation (SD). For each analyzed parameter, at least 15 plants per condition were measured within each independent biological replicate, resulting in a total of at least 45 plants analyzed per condition across the three replicates (n = 3).

With regard to the effects of the *L. riparium* extract, no differences were observed in root length compared to the control; a 30% increase in fresh shoot weight was detected at the 1% concentration (data not statistically significant) (**Figure 3B**).

#### 3.3.4 Salt stress experiment with by-product and extract of *T. barbieri*

In the case of *T. barbieri* under salt stress conditions, no marked differences were observed compared to the control when either the liquid by-product or the extract was applied (**Figure 3C, D**).

Regarding the extract of *T. barbieri*, the 3% concentration showed a tendency toward increased growth, with root length increasing by 54% and fresh weight of the aerial part by 39% compared to the control (data not statistically significant) (**Figure 3D**).

#### 3.3.5 Osmotic stress experiment with by-product and extract of *L. riparium*

Osmotic stress was simulated by adding mannitol 300 mM both in the treated plates and in their respective controls. Tobacco seedlings, treated with *L. riparium* liquid by-product, showed no differences in root growth. However, shoot fresh weight was 94% higher than the untreated control at the 3% treatment concentration (**Figure 4A**).

**Figure 4:**
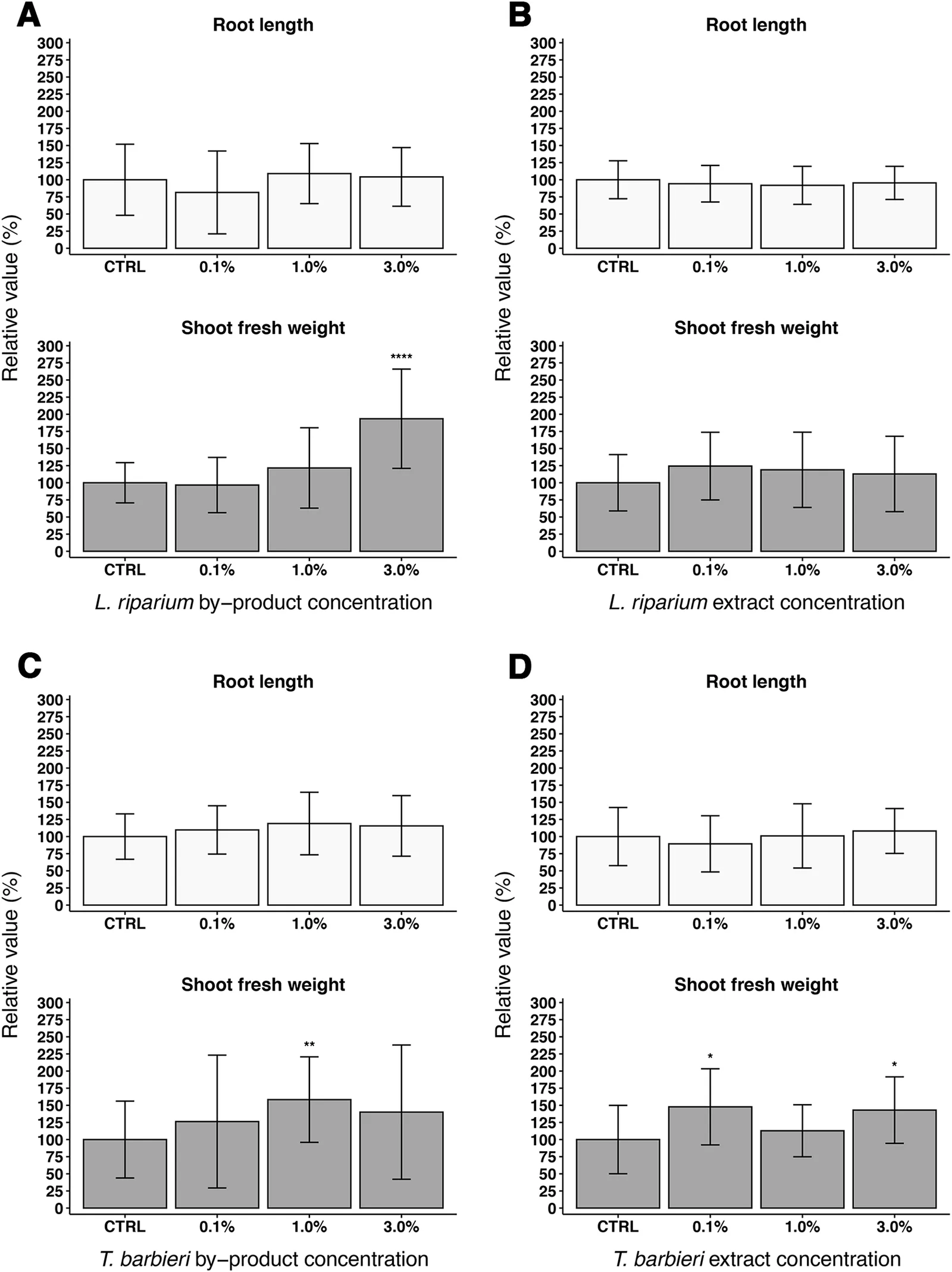
Evaluation of the biostimulant effects under osmotic stress condition of the treatment based on **(A)** by-product of moss *L. riparium*; **(B)** *L. riparium* moss extract; **(C)** by-product of moss *T. barbieri*; **(D)** *T. barbieri* moss extract, added at different concentrations, from 0.1 to 3%, to the culture medium. After 15 days of growth, the length of the primary root and the fresh weight of the aerial part of *N. tabacum* plants were measured. The data are expressed as relative (%) setting the control (CTRL) equal to 100. Statistical analysis was performed using Welch’s ANOVA, followed by Games–Howell *post hoc* tests to adjust p-values for multiple comparisons. Significance levels were indicated as follows: p < 0.05 (*), p < 0.01 (**), p < 0.001 (***), p < 0.0001 (****). In the Figure, only significant differences relative to the control group are displayed; the overall Welch’s ANOVA and the complete matrix of Games-Howell *post-hoc* pairwise comparisons are available in **Supplementary Data Sheet 1** (Sheet: **Figure 4**). Error bars displayed in the figures represent the Standard Deviation (SD). For each analyzed parameter, at least 15 plants per condition were measured within each independent biological replicate, resulting in a total of at least 45 plants analyzed per condition across the three replicates (n = 3).

The extract also showed positive effects on the growth of the aerial part of the plant with a positive trend of up to 24% in the highest concentration (data not statistically significant) (**Figure 4B**).

#### 3.3.6 Osmotic stress experiment with by-product and extract of *T. barbieri*

As regards the two derivatives of moss *T. barbieri* under osmotic stress conditions, no difference was found in root growth. The liquid by-product led to a 53% increase at 1% concentration in shoot fresh weight (**Figure 4C**) and also the extract increased this parameter by 48% at the 0.1% and by 43% at the 3% concentration (**Figure 4D**).

## 4 Discussion

Aquatic mosses are gaining attention for commercial use stimulating the development of new biomass cultivation approaches. When these slow-growing organisms are cultivated in environmentally exposed conditions, they accumulate extensive microbial contamination and may therefore act as vectors of biological contaminants. For this reason, moss mats are often washed and sterilized before use as decoration or filtering material. Live moss free of heavy biotic contaminations, produced for decoration in aquariums, is expensive due to complex cultivation procedures. Multiple applications, including biofiltration and bioproduction (Del Piano et al., 2026), require large quantities of axenic or semi-axenic viable biomass, but cultivation *in vitro* remains expensive for these, as for other slow-growing organisms. Since this kind of high-quality biomass and their by-products are a new product, this study explores the potential use of by-products from aquatic moss propagation as valuable natural plant biostimulants.

The production of biostimulants from industrial and agricultural by-products, within a circular economy framework, currently represents a promising solution for sustainable agriculture. Specifically, the development of biostimulants from “by-product” of the *in vitro* micropropagation process of plant organisms is a promising strategy (Ogunsanya et al., 2022). We focused on by-products derived from the propagation of two aquatic moss species, *L. riparium* and *T. barbieri*. They are cultivated without added phytohormones, but their cultivation *in vitro* has costs similar to classic *in vitro* micropropagation of other commercial plants.

These mosses, mostly produced for decoration in aquaria, have been studied for phytoremediation purposes and proposed as biofilters in multitrophic systems, aquaponics setups, and compact biofilter units (Papadia et al., 2020; Amitrano et al., 2025; Del Piano et al., 2026). As previously reported (Anglana et al., 2024), growth in Temporary Immersion Bioreactors (TIB) proved more advantageous than conventional liquid culture in flasks. This trend was observed in both species, but was more pronounced in *T. barbieri*, whose biomass increase in flasks was comparable to that of *L. riparium*, yet its Relative Growth Rate (RGR) in TIB was nearly twice as high (0.049 ± 0.008 g g^-1^ day^-1^) (**Figure 1A**).

This technology, introduced a few years ago for large-scale propagation of various plant species, offers several advantages: higher micropropagation yield, improved quality, reduced environmental impact, and pathogen-free plants under controlled *in vitro* conditions, making it suitable for advanced genetic improvement techniques (Georgiev et al., 2014). Given the clear improvement in moss propagation with TIB over traditional flask cultures, we tested both species in different growth media.

A previously reported key difference (Anglana et al., 2024) was confirmed: *L. riparium* grows more slowly in water alone (0.026 ± 0.005 g g^-1^ day^-1^) but performs better in nutrient media such as PpNO_3_, whereas *T. barbieri* thrives in nutrient-free conditions, showing markedly higher growth in deionized water (0.049 ± 0.008 g g^-1^ day^-1^) and reduced growth in enriched media such as PpNH_4_ (**Figure 1B**). This growth pattern in *T. barbieri* may be linked to selection pressures from prolonged cultivation in laboratory and aquarium environments under varying conditions, which has also resulted in the absence of sporophyte formation in this species (Wang and Jia, 2019; Anglana et al., 2024).

In any case, both species can be efficiently propagated in plain water or in media with a very low nutrient concentration (maximum 5%), representing an economic advantage and enabling the recovery of a by-product and, according to our preliminary estimates, representing an approach characterized by high resource-use efficiency.

A comprehensive phytohormonal characterization was performed in moss dry biomass, in the liquid by-product obtained after 30 days of growth in TIB systems, and in biomass-derived extracts of *L. riparium* and *T. barbieri*. This separation allowed us to distinguish between intracellular hormone pools, compounds released into the culture medium, and those recovered after extraction.

Bryophytes are known to synthesize a broad spectrum of phytohormones, as extensively documented for *P. patens* (Guillory and Bonhomme, 2021) and for multiple bryophyte taxa (Záveská Drábková et al., 2015). In the latter study, more than 40 phytohormones were identified across 30 species, including cytokinins, auxins, gibberellins, brassinosteroids, ABA, jasmonates, and salicylates.

Consistent with the literature, cytokinins and auxins were the predominant classes detected in our moss biomasses (**Tables 1**, **2**). Cytokinin profiles were characterized by a predominance of cis-zeatin (cZ) and isopentenyl-type (iP-type) cytokinins, in agreement with previous bryophyte studies. In particular the highest free cZ concentration was observed in *L. riparium* biomass (159.867 pmol g^-1^), markedly exceeding trans-zeatin (tZ) levels. A similar cZ > tZ pattern was found in *T. barbieri*, although at lower absolute concentrations. Among conjugated forms, cZOG reached 212.639 pmol g^-1 ^in *L. riparium*, whereas cZROG accumulated to 1027.489 pmol g^-1 ^in *T. barbieri*.

This predominance of cis-zeatin over trans-zeatin types mirrors the trend reported by Záveská Drábková et al. (2015) where cZ-type cytokinins exceeded tZ-types in the majority of analyzed species. Free tZ was present at relatively low concentrations, with the highest value detected in *L. riparium* biomass (0.921 pmol g^-1^). Other tZ-related forms were mainly conjugated derivatives.

Isopentenyladenine-type cytokinins (iP, iPR) were detected in comparable amounts in both species, confirming the relevance of iP-type cytokinins in bryophyte metabolism.

Regarding auxins in biomass (**Tables 1**, **2**), indole-3-acetic acid (IAA) was detected in both species, with the highest concentration in *T. barbieri* biomass (149.23 pmol g^-1^). However, the most abundant auxin-related compound overall was its primary catabolite, 2-oxindole-3-acetic acid (oxIAA), which reached 3168.67 pmol g^-1^ in *T. barbieri*. The predominance of oxIAA over IAA is consistent with previous findings in bryophytes (Záveská Drábková et al., 2015) and suggests active auxin turnover. In contrast to vascular plants—where amino acid conjugates such as IAA-Asp are common (Ludwig-Müller, 2011)—IAA conjugates were either absent or detected only at very low levels in our moss biomasses.

Abscisic acid (ABA), typically reported in bryophytes together with its catabolites, was not detected in biomass in our study, indicating either low intracellular levels or rapid turnover under the adopted growth conditions (**Tables 1**, **2**).

The liquid medium collected after 30 days of culture, by-product, represents a previously unexplored matrix in the context of bryophyte propagation. Unlike biomass, literature data on hormone concentrations in moss culture wastewater are currently unavailable.

In this liquid by-product free cZ was detected in both species, with higher levels in *L. riparium* (2.183 pmol mL^-1^); iP was present at very low concentrations and ABA was detected in both species, particularly in *L. riparium* (2.54 pmol mL^-1^) (**Tables 1**, **2**). The selective presence of free cytokinin bases (mainly cZ) and ABA in the medium suggests active secretion or passive release during growth. Although concentrations are lower than in extracts, these findings indicate that the liquid by-product retains measurable amounts of biologically relevant phytohormones, supporting its potential valorization.

Biomass extract resulted in the recovery and concentration of multiple cytokinin and auxin forms, often at levels markedly higher than those measured in the original dry biomass (expressed on a volume basis). The highest tZ concentration overall was found in *T. barbieri* extract (17.060 pmol mL^-1^). Extremely high levels of cZ derivatives were detected in *T. barbieri* extract, including cZROG (14782.133 pmol mL^-1^) and cZR (721.330 pmol mL^-1^) (**Tables 1**, **2**). cZR5’MP was detected exclusively in extracts, particularly in *L. riparium*, whereas DHZR was found only in extracts, mainly from *T. barbieri*. Isopentenyl-type cytokinins were also enriched in extracts: iP was higher in *L. riparium* extract, while iPR reached its highest value in *T. barbieri* extract (106.555 pmol mL^-1^) (**Tables 1**, **2**).

Overall, these results indicate that extraction did not simply result in higher detectable cytokinin levels, but revealed markedly different cytokinin profiles between the two species. *T. barbieri* was characterized by a strong predominance of cZ-type cytokinins, particularly cZROG and cZR, whereas *L. riparium* showed a comparatively greater contribution of specific forms such as iP and cZR5′MP. The occurrence of several cytokinin forms exclusively in the extracts further suggests that processing allowed the recovery or detection of compounds that were absent or below detectable levels in the original biomass. However, their appearance after extraction should not necessarily be interpreted as evidence of *de novo* formation, as it may also reflect differences in extractability, release from the biomass matrix, or processing-related changes.

Aromatic cytokinins (topolin ribosides: mTR, oTR, pTR) and kinetin were detected exclusively or predominantly in extracts, with kinetin concentrations exceeding 330 pmol mL^-1^in both species. The presence of different cytokinin forms in the extracts indicates that the recovered cytokinin pool was chemically heterogeneous rather than being represented exclusively by biologically active free bases.

In our measurements Kinetin was detected both as free base (K) and riboside (KR), indicating the coexistence of active and storage/transport cytokinin forms (**Tables 1**, **2**). Notably, both K and KR were detected only in the biomass extracts. Because these extracts were obtained through a high-temperature autoclave treatment, these compounds should be interpreted cautiously, and their detection cannot be taken, in the present dataset, as unequivocal evidence of endogenous accumulation in the starting biomass (Argueso and Kieber, 2024). Thus, although the extraction process clearly produced a cytokinin-rich fraction, the composition of this fraction should be regarded as the phytohormone profile of the processed extract rather than as a direct representation of the endogenous cytokinin pool of intact moss biomass.

Auxins (IAA, oxIAA) were also present in extracts, while IAAsp was detected only at low concentrations in this matrix. ABA was detected in both extracts, with slightly higher levels in *T. barbieri* (**Tables 1**, **2**). Although quantitatively less abundant than cytokinins, the simultaneous occurrence of IAA, its oxidative metabolite oxIAA, and ABA further indicates that the extracts contained a heterogeneous pool of phytohormone-related compounds. In particular, the distinction between IAA and its derivatives is relevant when considering the potential biological significance of the measured concentrations, since oxidation and conjugation are major routes involved in the metabolic inactivation and homeostasis of bioactive IAA (Casanova-Sáez et al., 2021; Mateo‐Bonmatí et al., 2021).

Overall, extracts contained a complex mixture of active cytokinin free bases, ribosides commonly associated with cytokinin transport and interconversion, biosynthetic riboside-5′-monophosphates, and reversible O-glucosylated conjugates, in both cis- and trans-configurations (Sakakibara, 2006; Romanov and Schmülling, 2022). O-glucosylation is generally considered a reversible cytokinin inactivation mechanism, with O-glucosides representing a potentially reactivatable storage pool through β-glucosidase-mediated hydrolysis (Chen et al., 2021). Therefore, the biochemical complexity observed in the moss extracts is important because the high total cytokinin concentration does not correspond exclusively to immediately bioactive forms but comprises compounds with different metabolic and functional roles. The highest overall levels of plant hormones, and particularly cytokinins, were detected in the extract of *T. barbieri*. Cytokinins play key roles in plant growth and development including the regulation of cell division and expansion, nutrient metabolism, chloroplast function and senescence (Sakakibara, 2006; Kieber and Schaller, 2018). They dominate the extract fraction by a wide margin with over 100-fold higher molar concentration than auxins in both species, mainly thanks to cis-zeatin conjugates (cZROG, cZR, cZR5’MP) and kinetin. The predominance of different cis-zeatin forms is noteworthy, as cytokinin pools comprise compounds with different metabolic roles, including free bases, ribosides, nucleotides and conjugated forms (Sakakibara, 2006; Romanov and Schmülling, 2022). Thus, rather than being represented by a single predominant form, the moss extracts contained a chemically diversified cytokinin pool. Cytokinin totals in the extracts (equivalent to 4493.6–18289.3 nM) are two to three orders of magnitude above the ~10 nM minimum threshold typically required for cytokinin-responsive phenotypic changes (Wally et al., 2013).

Auxins (IAA + oxIAA + IAAsp) sit in the tens-of-nM range in the extracts (48–78 nM), below the ~100 nM threshold commonly cited for auxin bioactivity in some cases and higher in others depending on which specific IAA fraction is considered active (Wally et al., 2013). The occurrence of IAA together with oxIAA and IAAsp also indicates the presence of different components of auxin metabolism, since oxidation and conjugation represent important pathways involved in IAA homeostasis (Casanova-Sáez et al., 2021; Mateo‐Bonmatí et al., 2021). For both phytohormones categories, the liquid by-products (spent culture medium) are far more dilute but nonetheless present. The reference concentration expressed in nM where possible, are reported in **Table 3** to facilitate comparison with literature.

**Table 3:** Phytohormone category totals (summed across all detected forms within each class), converted in ng/g or nM where possible, to facilitate comparison with data reported by most of literature.

Species	Matrix	Cytokinins (total)	Auxins (total)	ABA	Reference
*L. riparium*	biomass	124.3 ng/g	236.4 ng/g	ND	This work
*L. riparium*	by-product	2.3 nM	ND	2.5 nM	This work
*L. riparium*	extract	4493.6 nM	48.1 nM	0.6 nM	This work
*T. barbieri*	biomass	594.5 ng/g	631.1 ng/g	ND	This work
*T. barbieri*	by-product	0.6 nM	ND	0.7 nM	This work
*T. barbieri*	extract	18289.3 nM	77.7 nM	1.2 nM	This work
*Ascophyllum nodosum* extract	biomass	1–61.7 ng/g	6–615 ng/g	0–2 ng/g	Wally et al., 2013
*Ecklonia maxima*	biomass	0.0111–3 ng/g	9.7–22.2 ng/g	0–1 ng/g	Stirk et al., 2003; Wally et al., 2013
*Macrocystis* spp.	biomass	0.029–9.1 ng/g	14.6 ng/g	1 ng/g	Stirk et al., 2003; Wally et al., 2013
*Ulva* spp.	biomass	0.0242–14.9 ng/g	ND	ND	Stirk et al., 2003, 2009
*Dictyota* spp.	biomass	0.0191–29.7 ng/g	ND	ND	Stirk et al., 2003, 2009
*Sargassum* spp.	biomass	0.0263–30 ng/g	1091 ng/g	10 ng/g	Stirk et al., 2003; Wally et al., 2013
*Gigartina* spp.	biomass	0.0058–0.0103 ng/g	ND	ND	Stirk et al., 2003
*Sarcothalia* spp.	biomass	0.0078–0.018 ng/g	ND	ND	Stirk et al., 2003
*Amphiroa* spp.	biomass	0.0359–0.0458 ng/g	ND	ND	Stirk et al., 2003
*Caulerpa* spp.	biomass	0.0169–2.86 ng/g	15.41–53.6 ng/g	0.618 ng/g	Stirk et al., 2003; Yalçın et al., 2019
*Cladophora capensis*	biomass	0.0399 ng/g	ND	ND	Stirk et al., 2003
*Halimeda cuneata*	biomass	0.0118 ng/g	ND	ND	Stirk et al., 2003
*Aeodes orbitosa*	biomass	0.024 ng/g	ND	ND	Stirk et al., 2003
*Hypnea spicifera*	biomass	0.0557 ng/g	ND	ND	Stirk et al., 2003
*Mazzaella capensis*	biomass	0.0272 ng/g	ND	ND	Stirk et al., 2003
*Nothogenia erinacea*	biomass	0.0049 ng/g	ND	ND	Stirk et al., 2003
*Plocamium corallorhiza*	biomass	0.0236 ng/g	ND	ND	Stirk et al., 2003
*Carradoeriella virgata*	biomass	0.0284 ng/g	ND	ND	Stirk et al., 2003
*Porphyra capensis*	biomass	0.0587 ng/g	ND	ND	Stirk et al., 2003
*Suhria vittata*	biomass	0.0061 ng/g	ND	ND	Stirk et al., 2003
*Arthrocardia* sp.	biomass	0.0251 ng/g	ND	ND	Stirk et al., 2003
*Cheilosporum* sp.	biomass	0.0192 ng/g	ND	ND	Stirk et al., 2003
*Corallina* sp.	biomass	0.0191 ng/g	ND	ND	Stirk et al., 2003
*Jania* sp.	biomass	0.0117 ng/g	ND	ND	Stirk et al., 2003
*Cystoseira* sp. *f. verticillatum*	biomass	0.406 ng/g	3.53 ng/g	1.123 ng/g	Yalçın et al., 2019
*Cystoseira sinuosa*	biomass	2.86 ng/g	53.6 ng/g	ND	Yalçın et al., 2019
*Cystoseira barbata*	biomass	1.038 ng/g	15.41 ng/g	0.618 ng/g	Yalçın et al., 2019
*Halopteris incurva*	biomass	2.744 ng/g	11.808 ng/g	0.49 ng/g	Yalçın et al., 2019
*Gracilaria bursa-pastoris*	biomass	0.047 ng/g	0.48 ng/g	0.243 ng/g	Yalçın et al., 2019
*Ellisolandia elongata*	biomass	2.074 ng/g	3.98 ng/g	0.31 ng/g	Yalçın et al., 2019
*Padina scopulorum*	biomass	29.98 ng/g	0.82 ng/g	ND	Yalçın et al., 2019
*Peyssonnelia capitata*	biomass	8.01 ng/g	ND	0.12 ng/g	Yalçın et al., 2019
*Feldmannia petiolata*	biomass	0.229 ng/g	10.45 ng/g	0.196 ng/g	Yalçın et al., 2019

Commercial seaweed extracts, among the most common biostimulants, are well known to contain plant growth regulators, including trans-zeatin, trans-zeatin riboside, their dihydro derivatives, and predominantly zeatin (Z) and isopentenyl (IP) conjugates. They are also rich in auxins and auxin-like compounds (IAA) and water-soluble growth inhibitors such as ABA (Khan et al., 2009). Although moss- and seaweed-derived preparations represent different biological matrices, the coexistence of multiple cytokinin forms, auxins and ABA provides a useful qualitative basis for comparison. Anyhow the concentration found for these molecules seems to be too low to explain the biostimulant effect (Wally et al., 2013). This observation further supports the view that the biological activity of complex biostimulant preparations may depend on the combined action of different bioactive constituents rather than on individual phytohormones alone.

To test whether the processed moss biomass and liquid by-product from moss propagation can serve as valuable sources of these compounds, active in phytostimulation and comparable to seaweeds or other phytohormone-based biostimulants we evaluated the effects on growth *in vitro* of the model plant *N. tabacum.*

Preliminary *in vitro* assays on *N. tabacum* confirmed that both moss species, *L. riparium* and *T. barbieri*, produce biologically active derivatives with biostimulant potential. However, their performance varied markedly depending on the matrix (biomass extract vs liquid by-product) and on the growth conditions (optimal vs abiotic stress).

The formulations were tested at 0.1, 0.5, 1, 3 and 5% (v/v). Since they had not been previously evaluated *in vitro* and contained growth-regulating compounds, including auxin- and cytokinin-like substances, the concentration range was selected as an initial dose–response screening, starting from relatively low concentrations to minimize the risk of growth inhibition or phytotoxicity. This precaution was consistent with previous studies reporting concentration-dependent and frequently biphasic responses to plant biostimulants, in which low or intermediate doses stimulated growth whereas higher doses had neutral or inhibitory effects (Ugena et al., 2018; Ogunsanya et al., 2022; Yuan and Dickinson, 2024).

To the best of our knowledge, studies evaluating moss-derived extracts or culture by-products as plant biostimulants in comparable *in vitro* assays are currently very limited. Therefore, in the absence of directly comparable data for moss-derived products, the present results were primarily discussed in relation to algal and microalgal biostimulants, which represent more extensively investigated photosynthetic biomass-derived products and provide a useful framework for comparison.

Under standard conditions, the most effective treatment overall was the extract of *T. barbieri*. At the lowest concentration (0.1%), it significantly enhanced primary root length by up to 49% and shoot fresh weight by 93% compared to the untreated control (**Figure 2D**). The higher effectiveness observed at the lowest application rate indicates a marked concentration-dependent response, suggesting that relatively small amounts of the extract were sufficient to stimulate seedling growth, whereas increasing the concentration did not result in a proportional enhancement of the response. Similar non-linear and concentration-dependent responses have been reported for algal-derived biostimulants. Ghaderiardakani et al. (2019), for example, observed stimulation of *Arabidopsis thaliana* primary root growth by low concentrations (0.03–0.08%) of *Ulva intestinalis* extract, with approximately 80% stimulation at 0.06%, whereas concentrations ≥0.3% progressively inhibited root growth. Similarly, Shefer et al. (2022) reported a dose-dependent response of *A. thaliana* to a preparation derived from *Ulva*, with the most effective treatment promoting both root elongation and plant biomass. Although direct quantitative comparison is limited by differences in extract composition, concentration units and experimental conditions, these studies provide a useful reference for the pronounced response observed at the low concentration of *T. barbieri* extract and support the importance of application rate in determining the growth response to complex natural extracts.

The extract of *L. riparium* also showed relevant activity, with the best performance at 0.5%, increasing root length by 30% and fresh biomass by 78% (**Figure 2B**). Although slightly less effective than *T. barbieri* extract under these conditions, its stimulatory effect was consistent across parameters. Interestingly, the concentrations associated with the maximum response differed between the two moss species (0.1% for *T. barbieri* and 0.5% for *L. riparium*), indicating that biological activity was dependent not only on application rate but also on the source of the extract. Species- and extraction-dependent differences in biostimulant activity have also been observed among extracts obtained from photosynthetic microorganisms. Chovanček et al. (2023), for example, reported variable effects among microalgal extracts evaluated through an *A. thaliana* root-growth assay, depending on both the biological source and extraction procedure. In the present study, the different responses elicited by *T. barbieri* and *L. riparium* are consistent with the marked differences observed in their phytohormone profiles. However, this association should be interpreted cautiously, since the growth-promoting effects cannot be attributed exclusively to phytohormones and may involve other bioactive components of the extracts.

In contrast, the liquid by-products exhibited more moderate effects under optimal growth conditions. The spent culture medium of *L. riparium* induced increases in shoot biomass ranging from 18% to 26%, with the 1% concentration being the most effective (**Figure 2A**). The liquid by-product of *T. barbieri* showed more limited activity, except for a 51% increase in primary root length at the 3% concentration, while shoot biomass stimulation remained limited (+14%) (**Figure 2C**). The generally weaker response to the liquid by-products compared with the biomass extracts under standard conditions is consistent with their lower concentrations of the phytohormones quantified in the present study. Nevertheless, the measurable activity of these by-products indicates that the spent culture medium retained biologically active components despite representing a considerably more diluted matrix. This observation is consistent with evidence that extracellular metabolites released by photosynthetic microorganisms into their culture medium can exert plant growth-promoting effects. In particular, Moon et al. (2024) reported that cell-free culture supernatant from *Chlorella vulgaris* promoted root growth and shoot development in *A. thaliana*, providing evidence that culture media themselves can represent a source of biostimulant activity. Although the biological origin and composition of microalgal supernatants differ from those of moss culture media, these findings provide a useful comparative framework for interpreting the activity observed here in the spent culture media of *L. riparium* and *T. barbieri*.

Under salinity stress (150 mM NaCl), the performance ranking changed markedly. The liquid by-product of *L. riparium* showed the strongest stress-mitigating activity: at 3% concentration, root length increased by 260% and shoot fresh weight by 301% compared to the stressed control, and even the 1% treatment produced increases above 200% (**Figure 3A**). In contrast, the biomass extract of *L. riparium* displayed limited activity under salt stress, with no significant effects on root growth and only a slight, non-significant increase in shoot biomass at 1% (**Figure 3B**). The ability of algal- and microalgal-derived biostimulants to improve plant performance under salinity has been reported in different experimental systems and has been associated with multiple interacting mechanisms, including osmotic adjustment, antioxidant responses, ion homeostasis and hormonal regulation (El Boukhari et al., 2020; Sarsekeyeva et al., 2024).

The striking efficacy of the liquid by-product, which was not reproduced by the corresponding biomass extract, suggests that the stress-mitigating activity is strongly matrix-dependent. Compounds released, extracellularly transformed or progressively accumulated in the culture medium during moss cultivation may contribute to this response. Differences between the two matrices may also reflect their distinct chemical composition and processing history, including the possible alteration of heat-sensitive components during high-temperature extraction. However, the compounds responsible for the pronounced response under salinity cannot be identified from the present bioassay alone.

For *T. barbieri*, neither the liquid by-product nor the extract showed statistically significant improvements under salinity (**Figures 3C, D**). However, the 3% extract showed a positive trend, with root length increasing by 54% and shoot biomass by 39%, indicating partial stress mitigation, albeit weaker than that observed for *L. riparium* liquid by-product. Overall, the contrasting performance of the two moss species under the same stress conditions further supports a species- and matrix-dependent biostimulant effect, consistent with the variable responses reported for algal-derived biostimulants under salinity stress (El Boukhari et al., 2020; Sarsekeyeva et al., 2024).

Under simulated osmotic stress (300 mM mannitol), responses were more moderate but still species- and matrix-dependent. For *L. riparium* the liquid by-product at 3% increased shoot fresh weight by 94%, although no significant effect was observed on root growth (**Figure 4A**). The biomass extract induced only a positive trend (up to +24%) in shoot biomass (**Figure 4B**).

For *T. barbieri* the liquid by-product increased shoot fresh weight by 53% at 1% while the biomass extract enhanced shoot biomass by 48% at 0.1% and 43% at 3% (**Figures 4C, D**).

The maintenance or stimulation of shoot biomass under osmotic stress is consistent with the stress-mitigating effects reported for algal-derived biostimulants under water-limiting conditions. In *A. thaliana*, *A. nodosum* extracts have been shown to improve plant performance under drought through physiological and molecular responses involving maintenance of photosynthetic performance, stomatal regulation, antioxidant responses and stress-related gene expression (Santaniello et al., 2017; Rasul et al., 2021). Similarly, Moon et al. (2024) reported improved drought tolerance in *A. thaliana* treated with cell-free culture supernatant from *C. vulgaris*. Although these studies employed drought or water-deficit conditions rather than mannitol-induced osmotic stress, they provide a useful comparative framework for the growth-promoting effects observed here. The latter study is also particularly relevant to the present results, as it supports the potential contribution of extracellular metabolites accumulated in culture media to stress mitigation, although the composition and biological origin of the two matrices are different.

Unlike salinity stress, osmotic conditions did not produce the pronounced recovery observed with *L. riparium* liquid by-product under NaCl treatment. However, both derivatives of *T. barbieri* maintained moderate growth-promoting activity. The different responses observed under salinity and osmotic stress are consistent with the distinct physiological effects imposed by these two conditions and further indicate that the effectiveness of the moss-derived products depends not only on species and matrix, but also on the type of stress.

Overall, *T. barbieri* extract was the most effective treatment under optimal growth conditions, whereas *L. riparium* spent culture medium showed the strongest biostimulant effect under salt stress. Under osmotic stress, responses were more moderate and distributed across both species and matrices, further highlighting the species-, matrix- and stress-dependent nature of the observed biostimulant activity.

These findings align with the general understanding that plant extracts, being complex mixtures of phytohormones and metabolites, can exert substantial biostimulant effects, although their effectiveness depends on the biological source, composition, concentration, and application conditions (du Jardin, 2015). These extracts trigger a range of physiological responses in plants, enhancing growth and resilience against biotic and abiotic stress. Due to the presence of numerous bioactive compounds, the observed effects cannot be attributed to a single component (Xu et al., 2020); fractionation studies confirm that no single fraction can replicate the full spectrum of effects of the complete extract, indicating that multiple constituents act independently or synergistically to elicit beneficial plant responses (Caradonia et al., 2019; Arencibia et al., 2022; Bisht and Chhabra, 2024). In the case of aquatic mosses, the high abundance of cytokinins represents a potentially relevant biochemical feature; however, total phytohormone concentration cannot be directly equated with biological activity because the detected pool includes compounds with different metabolic and functional roles. Nevertheless, the concentrations measured here provide an interesting basis for comparison with the phytohormone levels reported for commercial seaweed extracts (Wally et al., 2013). To the best of our knowledge, studies assessing moss-derived extracts and culture by-products as plant biostimulants in comparable experimental systems are currently very limited, while existing research on aquatic mosses has mainly focused on allelopathic effects (Nozaki et al., 2007; Kato-Noguchi et al., 2010). In the absence of directly comparable studies on moss-derived products, seaweed and microalgal extracts therefore provide the closest and most extensively investigated framework for comparison. Their biostimulant activity has historically been associated with phytohormones and other low-molecular-weight compounds (Mattner et al., 2013; du Jardin, 2015; Nayak et al., 2019; Arencibia et al., 2022). However, evidence from commercial seaweed extracts indicates that their phytohormone concentrations alone are generally insufficient to explain the observed biological effects at recommended application rates, suggesting that other constituents and the modulation of endogenous plant hormone pathways also contribute to their activity (Wally et al., 2013). Within this comparative framework, the present study extends the investigation of photosynthetic biomass-derived biostimulants to aquatic mosses and their culture by-products obtained through *in vitro* propagation systems.

Plant propagation and the production of bioactive compounds are relevant across multiple sectors, including pharmacology, nutraceutics, functional foods, cosmetics, and agriculture. TIB systems not only enable large-scale plant propagation but also facilitate the accumulation of secondary metabolites secreted into the culture medium. The resulting by-product, including discarded biomass and liquid culture medium, may therefore represent potentially valuable resources rather than waste, as they may contain bioactive molecules depending on the plant species. Medium enrichment depends on several parameters, including container design, immersion time and frequency, substrate composition, and elicitor use (De Carlo et al., 2021).

In the evolving field of agricultural biostimulants, intellectual property (IP) rights play a crucial role in protecting innovations related to ingredients, production techniques, formulations, and applications (Verma and Manhas, 2024). Due to their biological nature, biostimulants face unique challenges in IP protection, but tools like patents, trademarks, and trade secrets provide legal safeguards and promote product commercialization. Green Greener srl (Policoro, Italy) was granted the Italian patent No. 102024000012364, on 18/05/2026 (Pending European application EP4656050, published on 03/12/2025) titled “Method for the production of organic plant stimulants” based on the practical evidence of the beneficial effects on plants of liquid by-product obtained by aquatic mosses cultivation. This method overcomes limitations of conventional hormone extraction, chemical synthesis, and microbial fermentation, offering a sustainable alternative for plant biostimulation. Here we studied analogous situation confirming the phytostimulant effects and associating them to a significant concentration of phytohormones.

Interestingly, irrespective of their direct contribution to the observed biostimulant effects, the phytohormone levels detected in the moss-derived preparations were in several cases comparable to, or higher than, concentrations reported for seaweeds and seaweed-derived extracts (Stirk et al., 2003, 2004, 2009; Wally et al., 2013; Yalçın et al., 2019, 2020). This comparison, summarized in **Table 3**, highlights aquatic moss biomass and its derived products as a potentially interesting source of phytohormones for further investigation, including their possible recovery and concentration.

## 5 Conclusion

Moss cultures propagated in temporary immersion bioreactors produce bioactive metabolites including growth-regulating phytohormones and release part of them into the growth medium. In this study, we identified their molecular forms in both the biomass and the secreted fraction. *In vitro* bioassays confirmed the biostimulant potential of these derivatives. Treatments based on individual components, including propagation liquid by-products and biomass-derived extracts, induced measurable morphological changes in treated plants.

Chemical analyses revealed the presence of various phytohormones, even at low concentrations, suggesting that these compounds contribute to the observed biological effects. This is consistent with the known mechanisms of microalgae-based biostimulants, which are able to exert physiological activity even at low doses (Wally et al., 2013; Rupawalla et al., 2022) but in this case of study, phytohormones concentration reach and overcome the thresholds known for bioactivity of purified forms.

Among the tested preparations, the spent culture medium of *L. riparium* showed a particularly pronounced activity under salinity, substantially restoring root growth and shoot biomass in NaCl-stressed plants. This stress-dependent activity distinguishes the liquid by-product from the biomass extracts and identifies *L. riparium* exudates as a particularly promising material for the development of biostimulants aimed at improving plant performance under saline conditions.

Overall, moss-derived propagation water (liquid by-product) and biomass waste represent cost-effective, technically feasible, and sustainable resources for developing innovative plant biostimulant products, alternative to the more popular algae farming, contributing to the valorization of a new bio resource within a circular production framework.

## Data Availability

The raw data supporting the conclusions of this article will be made available by the authors, without undue reservation.
